# Neuropsychological Assessment of Community-Dwelling Older Adults in Almaty, Kazakhstan

**DOI:** 10.3390/ijerph192316189

**Published:** 2022-12-03

**Authors:** Mukhtar Kulimbet, Natalya Glushkova, Beth Snitz, Radmila Tsoy, Shalkar Adambekov, Evelyn Talbott, Alibek Mereke, Minjie Wu, Abzal Zhumagaliuly, Ferhat Karaca, Yuefang Chang, Saule Turuspekova, Akira Sekikawa, Kairat Davletov

**Affiliations:** 1 Department of Epidemiology, Biostatistics and Evidence-Based Medicine, Al-Farabi Kazakh National University, Almaty 050040, Kazakhstan; 2 Health Research Center, Asfendiyarov Kazakh National Medical University, Almaty 050000, Kazakhstan; 3 Department of Neurology, University of Pittsburgh, Pittsburgh, PA 15237, USA; 4 Department of Nervous Diseases, Asfendiyarov Kazakh National Medical University, Almaty 050000, Kazakhstan; 5 Department of Epidemiology, School of Public Health, University of Pittsburgh, Pittsburgh, PA 15213, USA; 6 Department of Psychiatry, University of Pittsburgh, Pittsburgh, PA 15213, USA; 7 Public Health Department, Asfendiyarov Kazakh National Medical University, Almaty 050040, Kazakhstan; 8 Department of Civil and Environmental Engineering, The Environment & Resource Efficiency Cluster, School of Engineering and Digital Sciences, Nazarbayev University, Nur-Sultan 010000, Kazakhstan; 9 Department of Neurosurgery, School of Medicine, University of Pittsburgh, Pittsburgh, PA 15213, USA

**Keywords:** cognitive impairment, dementia, neuropsychological test battery, normative data, Kazakhstan, older adults

## Abstract

Cognitive impairment in older adults is a major public concern for Kazakhstan’s aging population. We aimed to (1) administer a neuropsychological test battery (NTB) in domains relevant to aging-associated cognitive impairment in a sample of adults aged 60+ without dementia in Almaty, Kazakhstan; (2) investigate the associations between demographic factors and test performance; and (3) provide information on the distribution of NTB scores as preliminary local normative data relevant for this population. A cross-sectional evaluation of 276 participants aged 60+ in Almaty, Kazakhstan, was conducted using cognitive instruments including tests of memory, attention, language, executive functions, visuospatial abilities, and processing speed. Multiple linear regression analyses were used to examine the association of demographic factors with neuropsychological test performance. The results from the regression analysis showed that those who are younger, have more years of education, are women, and are of Russian ethnicity had significantly better performance. The current study illustrated (1) the feasibility of administering the NTB to older adults in the general population in Kazakhstan; (2) the preliminary local normative neuropsychological measures; and (3) their independent associations with age, education, gender, and ethnicity. The findings are a platform for future research on dementia and cognitive impairment in older adults in Kazakhstan.

## 1. Introduction

Kazakhstan is an upper-middle-income country, the second largest of the former Soviet republics in Central Asia, undergoing a rapid epidemiological transition [1]. Kazakhstan is expected to soon face a considerable cognitive impairment and dementia burden as the number of older adults aged 60+ is expected to increase by 70% between 2010 and 2030 and by 260% between 2010 and 2050 [2]. Moreover, the burden of cardiovascular disease, which is also a risk factor for cognitive impairment and dementia [3], is very high [4]. In addition, the population of Almaty, the largest city, has been exposed to high levels of air pollution [5,6], which is now regarded as a potentially modifiable risk factor for dementia [7].

Despite the anticipated rise in cognitive impairment and dementia in Kazakhstan, reports on the prevalence of cognitive impairment and dementia in Kazakhstan are scarce [2,8]. Recently, we have conducted a population-based study of older adults in Almaty, Kazakhstan, to estimate the prevalence of mild cognitive impairment (MCI) [9]. MCI diagnosis was made using published consensus criteria [3] by a multidisciplinary team of neurologists and psychiatrists in Kazakhstan. This was the first study that reported the prevalence of MCI in Kazakhstan. As in many epidemiological studies on cognition, we used a summary score from a brief global cognitive screening measure, the Mini-Mental State Examination (MMSE), as an initial screening and then administered more comprehensive tests to assess memory, attention, language, executive functions, visuospatial abilities, and processing speed. This is because global cognitive screening measures have limited sensitivity and do not capture varying levels of change that can occur across different cognitive domains relevant to aging-associated cognitive impairment [10]. However, most of the cognitive tests used in this study were available only in former Soviet Union countries.

In Kazakhstan, many comprehensive neuropsychological tests used in the US (e.g., Alzheimer’s Disease Neuroimaging Initiative [11]) are available in Russian, and these tests have been used in clinical settings [12,13,14]. To our best knowledge, no previous study has reported the distribution of the neuropsychological test scores and their associations with demographic factors in a Kazakh population of older adults without dementia. These data would help establish normative values for further research studies investigating the risk for cognitive impairment in Kazakhstan.

Therefore, the aims of this study were (1) to administer a neuropsychological test battery (NTB) in domains relevant to aging-associated cognitive impairment to a random sample of older adults aged 60 and older without dementia in Almaty, Kazakhstan; (2) to investigate the associations between demographic factors and test performance; and (3) to provide information on the distribution of NTB scores as preliminary local normative data relevant for this population.

## 2. Materials and Methods

We conducted a cross-sectional study among older adults of both genders aged 60 years and older who had lived in Almaty for the last 20 years. We recruited participants from an outpatient clinic. An outpatient clinic in Kazakhstan is a primary care facility to receive government-provided medical care. Every citizen in Kazakhstan must register at an outpatient clinic. We used outpatient clinic #32 in the Turksib District of Almaty, Kazakhstan. The Turksib District has historically been known as an older part of Almaty with a population of about 235,000. Migration within and between cities in Kazakhstan is uncommon due to cultural practices resulting in residents living in a certain area for decades. Kazakh and Russian are the major ethnic groups in Kazakhstan. Under the Soviet Union (1936–1991), the Kazakh language in school was banned and children uniformly learned Russian [15]. Thus, older adults in Almaty are uniformly fluent in Russian.

Participants had to meet the following criteria in addition to the age and residency criteria above: (1) fluency in speaking and writing the Russian language; (2) capacity to provide informed consent; and (3) an MMSE score of 23 or higher. Participants were excluded if they had significant neurological and mental disorders (e.g., Parkinson’s disease, major depression, etc.), severe health conditions (e.g., cancer, liver cirrhosis, renal failure, etc.), history of vascular surgery in the past 6 months (any surgery that used cardiopulmonary bypass), severe sensory (visual acuity or hearing loss) or language impairment which would compromise evaluation of cognitive status, prior history of head trauma with a loss of consciousness, an illness associated with excessive alcohol consumption, and any substance abuse or use of medications for sleep disorders for the last 30 days before the neuropsychological assessment.

We recruited participants using the Population Registration Portal, a national electronic patient management database with health information on each registered citizen of Kazakhstan. We built a de-identified list of subjects by age and predetermined health conditions based on the above-described inclusion and exclusion criteria. Using this list, we randomly selected potential participants. These potential participants were invited to the clinic and were screened by administering the MMSE. Written informed consent was obtained before the administration of MMSE. The NTB was administered only to those whose MMSE score was ≥23. We started recruitment in September 2019, and our target recruitment number was 400. However, due to the COVID-19 pandemic, we terminated our recruitment in March 2020 when we had screened 289. Among these 289 subjects, 276 participated in the study, resulting in a rate of participation of 95.5%. During the clinic visit, we collected information on gender, ethnicity (Kazakh and other central Asians vs. Russians and other Europeans), date of birth, and years of education.

### 2.1. Neuropsychological Test Battery (NTB)

A US neuropsychologist (BS) trained a Kazakh behavioral neurologist (RT) who is fluent in English, Russian, and Kazakh on neuropsychological assessment with older adults across a spectrum of cognitive status. The training was held in Pittsburgh, US, for three days. Then, these two neuropsychologists trained two fellows at the Kazakh National Medical University, who were fluent in English, Russian, and Kazakh. The behavioral neurologist (RT) and the two fellows administered the NBT.

We adopted the NTB used in the Duke–Tomsk PREPARE Study of the Duke University Alzheimer Disease Research Center in the US in conjunction with the Nebbiolo Center for Clinical Trials [12]. Russian-translated tests were back-translated into English to check the accuracy of the Russian translation. To attain interpretive equivalence, Russian translations were reviewed and revised by Russian specialists in psychology and medicine to ensure all items were appropriate, clearly worded, and could be readily understood by potential study participants [12]. Validation work has also been conducted including criterion and discriminant validity of the cognitive measures (healthy controls vs. patients with Alzheimer’s disease), using logistic regression, discriminant analysis, and Receiver Operating Characteristic analysis. Measures of equivalence were examined in relation to normative values from the English-speaking population in the US [16].

All participants were assessed individually in a quiet room in the outpatient clinic with comfortable settings to avoid distractions. Trained research interviewers administered face-to-face neuropsychological tests to participants, assessing executive functions/working memory, attention/psychomotor speed, verbal episodic memory, and language abilities.

MMSE [17] is a cognitive status test widely used to screen for low cognitive function and dementia. MMSE consists of 11 items grouped into five cognitive domains: orientation; registration; attention calculation; recall; and language, including visual construction.

Participants took part in a face-to-face interview with a standardized questionnaire. The first stage consisted of screening out dementia with the MMSE (cutoff point <23) and assessing potential risk factors. When participants passed the MMSE screen, the following tests were administered.

Questions on functional status were obtained by the Alzheimer’s Disease Cooperative Study (ADCS) cognitive function screening instrument about difficulties in daily life (e.g., controlling financial affairs, shopping, using the bus or metro system, eating, dressing, and washing) [18]. Other questions covered everyday cognition (ease of remembering a relative’s birthday, ability to understand and discuss TV shows, or currently read books), social interaction (taking part in family parties), and hobbies.

Global cognitive status was assessed using the Montreal Cognitive Assessment (MoCA) [19]. The Russian version of MoCA was validated [20]. The MoCA is a 30-point test assessing verbal learning and memory, visuospatial abilities, executive functions, attention/vigilance, confrontation naming verbal fluency, working memory, and orientation to time and place.

Additionally, participants were assessed by the Consortium to Establish a Registry in Alzheimer’s Disease (CERAD) Word List Learning test, including delayed recall and recognition [21], phonemic and semantic verbal fluency [22], Trail Making Test (TMT) A and B [23], and the Multilingual Naming Test (MINT) [24].

The CERAD Word List Learning test of verbal episodic memory involves three repeated learning trials of a ten word list, with immediate free recall in each trial, a 30 min delayed free recall, and a recognition condition with the original ten words and ten distractor words.

In semantic verbal fluency, participants were asked to say as many words as possible within 60 s from each of two categories: animals and vegetables. The total score was the number of correct words generated, with errors or repetitions subsequently excluded. In phonemic verbal fluency, participants were asked to say as many words as possible within 60 s for each of the letters (/A/), (/Π/), and (/C/), excluding proper nouns and same-word variations.

TMT A and B assess attention/psychomotor speed and executive functions, respectively. Participants are asked to connect a series of numbers and letters on a page with a pencil. TMT A is composed of the numbers 1 through 25. TMT B consists of numbers and letters and requires that subjects alternate between numbers and letters sequentially.

The multilingual naming test (MINT) is a confrontation naming test designed for bilingual speakers. Participants were shown and asked to name 32 black and white line drawings of objects. The score reflects the number of spontaneously correctly named objects (without cues).

The study was approved by the Institutional Review Boards of the Kazakh National Medical University and the University of Pittsburgh. Written informed consent was obtained from each participant.

### 2.2. Statistical Analysis

Statistical analysis was performed using SAS version 9.4 (SAS Institute Inc., Cary, NC, USA). The study subjects were categorized according to age <65 vs. ≥65 and years of education (<12 years vs. ≥12 years). Categorical variables were expressed as frequency and percent. We used the Chi-square and Fisher’s exact tests to assess differences in categorical variables. Scores of NTB were expressed as the median and interquartile range (25th and 75th percentile). The Mann–Whitney U test was used to compare the distribution by age group, gender, and ethnicity. Multiple linear regression analyses were performed to determine the association of age as a continuous variable, gender, ethnicity, and years of education with the neuropsychological test scores. Scores with a skewed distribution (e.g., errors) were log-transformed. A two-sided *p*-value < 0.05 was considered statistically significant.

## 3. Results

The mean age was 64.7 years (SD = 4.6). The overall and age-group-specific characteristics of the participants are presented in Table 1. Most of the study participants (57.6%) were in the younger age group (60–64 years old), and 67.0% were female. The majority (57.6%) had more than 12 years of education. The mean years of education was 13.1 (SD = 2.7). Russians and other Europeans accounted for 58.7%, and Kazakhs and other central Asian accounted for (41.3%). There was no statistically significant difference between age groups by gender (*p* = 0.289) and education (*p* = 0.216). However, ethnicity (*p* = 0.016) differed significantly between age groups (Table 1).

Table 2, Table 3, Table 4, Table 5 and Table 6 show the distribution of the NBT scores overall (Table 2), by gender (Table 3), by age group (Table 4), by ethnicity (Table 5), and by education (Table 6). In most tests, females had significantly better scores than males (Table 3). Exceptions included the MINT test (*p* = 0.098). In almost all tests, the younger age group (those aged < 65) had significantly better scores than the older age group (those aged ≥ 65) (Table 4). Exceptions included semantic fluency: animals test (*p* = 0.181). Russians and other Europeans had significantly better scores in almost all tests than Kazakhs and Central Asians. Exceptions included CERAD delayed recall (*p* = 0.728) (Table 5). In most tests, those with years of education ≥12 years had better scores than those with years of education <12 years (Table 6).

Table 7 presents the results of the multiple linear regression models to examine the association of age, gender, ethnicity, and education on neuropsychological test scores. Each category of age, years of education, gender, and ethnicity was significantly associated with almost all test scores. The older the participants were, the poorer their performance was. Likewise, the lower the years of education were, the poorer the performance was. Females had significantly better scores than males, and Russians and other Europeans had significantly better scores than Kazakh and Central Asians on almost all tests.

## 4. Discussion

Most of the currently used neuropsychological tests were developed and validated in developed countries [25]. Evidence is clear that the same normative data from one population cannot be applied to other populations without errors due to biases [26], as many unique socio-cultural and language factors influence performance on cognitive tests. In this study, we administered neuropsychological tests widely used in the US to a randomly selected sample of 276 older adults without dementia in Almaty, Kazakhstan. We observed that those with younger ages, those with greater years of education, women, and those with Russians and other European ethnicities had significantly better performance in the NTB. This study generated preliminary local normative data for this NTB.

Consistent with the sizeable neuropsychological literature on aging, performance on most NTB tests was negatively associated with age and positively associated with education [27]. Gender differences are less ubiquitous in the literature, although there is evidence for female advantage in verbal tasks, including verbal episodic memory [28]. Even though the association of years of education with the CERAD delayed recall test was not significant, after adjusting for age and gender, it became significant, indicating that the higher the education, the better the performance. This finding was consistent with previous studies [29,30], including ours [31]. Age was significantly associated with most tests, which is consistent with previous studies [30,32], including ours [31]. When we examined the gender difference in univariate analyses, women performed better than men in all scores except for CERAD word recognition, TMT A errors, TMT B errors, and MINT. However, after adjusting for age, years of education, and ethnicity, women performed better only in CERAD delayed recall, MINT, semantic fluency, ADCS, and some NTB tests. Non-significant difference by gender after adjusting for age, years of education, and ethnicity is partly because women were younger, had more education, and were more likely to be of Russian or other Caucasian ethnicity. Generally, the current study showed that being older and male and having lower education were associated with poorer NTB performance on almost all neuropsychological tests. This finding is consistent with previously reported studies in Australia, Colombia, Korea, and European countries [33,34,35,36]. Although our multiple linear regression analyses showed a significant difference in NTB performance by ethnicity, the reasons for this difference remained unknown. A future study with much larger sample is warranted.

There are several limitations of this study. First, all the participants were recruited from one outpatient clinic. Thus, the generalizability was limited. Second, the total number of participants was relatively small (*n* = 276) due to the COVID-19 pandemic, although we intended to recruit 400 participants. Finally, the number of male participants was much smaller than that of females. Thus, the results in males must be interpreted cautiously. Future research will benefit from including individuals from diverse regions and backgrounds and a larger sample size with more balanced proportions of males and females.

There are several strengths in this study. First, we used the NTB widely used in the US, which was translated and validated in Russian [12]. Second, the rate of participation was high: 95.5%, which ensured the generalizability of the result to the target population. Finally, this is the first study that collected local cognitive test performance ranges for Kazakhstan’s older adult population without dementia. We provided preliminary normative data stratified by gender, age group, and ethnicity for standard neuropsychological measures.

## 5. Conclusions

This study in Kazakhstan demonstrated the feasibility of administering the NTB widely used in neuropsychological research in the US and reported preliminary local normative neuropsychological measures and their independent associations with age, education, gender, and ethnicity. The findings are a platform for future research on dementia and cognitive impairment in older adults in Kazakhstan.

## Figures and Tables

**Table 1:** Demographic characteristics of study participants.

Characteristics		60–64 Years	≥65 Years	Total	*p*-Value
Gender	Female	110 (69.6)	75 (63.6)	185 (67.0)	0.289
	Male	48 (30.4)	43 (36.4)	91 (33.0)	
Education	>12 years	86 (54.4)	73 (61.9)	159 (57.6)	0.216
	≤12 years	72 (45.6)	45 (38.1)	117 (42.4)	
Ethnicity	Kazakh and Central Asian	78 (49.4)	36 (30.5)	114 (41.3)	0.002
	Russian and European	80 (50.6)	82 (69.5)	162 (58.7)	

Values represent numbers (proportion) within the age stratum and within the total sample.

**Table 2:** Summary statistics of neuropsychological test scores.

Neuropsychological Test Battery	Mean (SD)	Q_25_	Median	Q_75_
MOCA/30	23.8 (3.3)	22	24	26
CERAD learning trials, sum/30	17.0 (4.5)	14	17	20
CERAD delayed recall/10	4.37 (2.3)	3	5	6
CERAD word recognition: correct hits/10	8.5 (1.9)	8	9	10
CERAD word recognition: correct rejections/10	8.5 (0.8)	9	10	10
TMT A, time (s) *	59.0 (26.7)	69	51	40
TMT A errors	0.1 (0.4)	0	0	0
TMT B, time (s) *	150.3 (67.4)	194	132	97
TMT B errors	1.4 (1.8)	0	1	2
MINT/32	26.9 (4.0)	25	28	30
Semantic fluency: animals (no. words)	15.0 (4.8)	11	15	18.5
Semantic fluency: vegetables (no. words)	10.5 (3.5)	8	10	12
Phonemic verbal fluency (no. words, sum of 3 trials)	27.4 (9.8)	20	26	34
ADCS/max score/x *	3.1 (2.6)	1	3	4.5

Q_25_ represents the 25th percentile and Q_75_ represents the 75th percentile; SD—standard deviation; MOCA—Montreal Cognitive Assessment; CERAD—the Consortium to Establish a Registry in Alzheimer’s Disease; MINT—Multilingual Naming Test; TMT A—Trail making test A; TMT B—Trail making test B; Semantic animals and vegetables—Semantic verbal fluency; ADCS—ADCS mail-in cognitive function screening instrument; * Log transformation used in the modeling for TMT A time, TMT B time, and ADCS variables.

**Table 3:** Summary statistics for neuropsychological test scores for gender groups.

Variables	Gender						Test of Difference	
	Females			Males			U-Test *	*p*-Value
	Med	Q_25_	Q_75_	Med	Q_25_	Q_75_		
MOCA/30	25	22	26	23	21	26	6384.5	0.001
CERAD learning trials, sum/30	18	14	21	15	13	19	6683.5	0.005
CERAD delayed recall/10	5	3	6	3	2	5	6199.5	<0.001
CERAD word recognition: correct hits/10	9	8	10	9	7	10	7229.0	0.048
CERAD word recognition: correct rejections/10	10	9	10	10	9	10	7378.0	0.050
TMT A, time (s)	49	64	40	55	74	45	6810.0	0.010
TMT A errors	0	0	0	0	0	0	8136.0	0.403
TMT B, time (s)	123	182	94	141	217	99	7148.0	0.042
TMT B errors	1	0	2	1	0	2	8028.5	0.515
MINT/32	28	25	29	29	26	30	7399.0	0.098
Semantic fluency: animals (no. words)	16	12	19	14	10	17	6890.5	0.014
Semantic fluency: vegetables (no. words)	11	9	13	9	7	11	5216.0	<0.001
Phonemic verbal fluency (no. words, sum of 3 trials)	28	21	36	24	18	32	6941.5	0.018
ADCS/max score/x	3	2	4	2	1	4	6740.0	0.007

Med represents median. Q_25_ represents the 25th percentile and Q_75_ represents the 75th percentile. MOCA—Montreal Cognitive Assessment; CERAD—the Consortium to Establish a Registry in Alzheimer’s Disease; MINT—Multilingual Naming Test; TMT A—Trail making test A; TMT B—Trail making test B; ADCS—ADCS mail-in cognitive function screening instrument; * Mann–Whitney U test.

**Table 4:** Summary statistics for neuropsychological test scores for age groups.

Variables	Age Groups						Test of Difference	
	60–64 Years			≥65 Years			U-Test *	*p*-Value
	Med	Q_25_	Q_75_	Med	Q_25_	Q_75_		
MOCA/30	25	22	27	23	21	26	6774	0.002
CERAD learning trials, sum/30	18	15	21	15	12	18	5709.5	<0.001
CERAD delayed recall/10	5	3	6	4	2	5	6138.5	<0.001
CERAD word recognition: correct hits/10	9	8	10	9	7	10	7252.5	<0.001
CERAD word recognition: correct rejections/10	10	9	10	10	9	10	9102.0	0.693
TMT A, time (s)	49	61	40	57	84	42	6973.5	0.006
TMT A errors	0	0	0	0	0	0	8560	0.636
TMT B, time (s)	120	170	91	162	222	107	6460.5	<0.001
TMT B errors	1	0	2	1	0	2	7310	0.02
MINT/32	28	26	30	27	24	29	7318	0.025
Semantic fluency: animals (no. words)	15	12	19	14	11	18	7874.5	0.181
Semantic fluency: vegetables (no. words)	10	8	13	10	8	12	7422.5	0.04
Phonemic verbal fluency (no. words, sum of 3 trials)	28	21	36	24	17	33	6998	0.007
ADCS/max score/x	3	1	4	3	2	5	7230	0.018

Med represents median. Q_25_ represents the 25th percentile and Q_75_ represents the 75th percentile. MOCA—Montreal Cognitive Assessment; CERAD—the Consortium to Establish a Registry in Alzheimer’s Disease; MINT—Multilingual Naming Test; TMT A—Trail making test A; TMT B—Trail making test B; ADCS—ADCS mail-in cognitive function screening instrument; * Mann–Whitney U test.

**Table 5:** Summary statistics for neuropsychological test scores for ethnic groups.

Variables	Ethnicity Groups						Test of Difference	
	Kazakh and Central Asian			Russian and European			U-Test *	*p*-Value
	Med	Q_25_	Q_75_	Med	Q_25_	Q_75_		
MOCA/30	22	20	25	25	23	27	5174.5	<0.001
CERAD learning trials, sum/30	15	12	20	18	15	21	7142.5	<0.001
CERAD delayed recall/10	5	2	6	4	3	6	9008.5	0.728
CERAD word recognition: correct hits/10	9	7	10	9	8	10	8167.5	0.090
CERAD word recognition: correct rejections/10	10	9	10	10	9	10	8551.5	0.219
TMT A, time (s)	56	85	45	48	64	40	6977.0	0.001
TMT A errors	0	0	0	0	0	0	7770.5	<0.001
TMT B, time (s)	153	226	105	122	170	89	6917.0	<0.001
TMT B errors	1	0	2	1	0	2	7632.0	0.011
MINT/32	26	23	28	29	27	30	5398.5	<0.001
Semantic fluency: animals (no. words)	13	10	16	16	13	20	5971.0	<0.001
Semantic fluency: vegetables (no. words)	9	7	12	11	9	13	6338.0	<0.001
Phonemic verbal fluency (no. words, sum of 3 trials)	24	19	32	29	22	36	7128.5	0.001
ADCS/max score/x	3	1	5	3	1	4	8908.0	0.613

Med represents median. Q_25_ represents the 25th percentile and Q_75_ represents the 75th percentile. MOCA—Montreal Cognitive Assessment; CERAD—the Consortium to Establish a Registry in Alzheimer’s Disease; MINT—Multilingual Naming Test; TMT A—Trail making test A; TMT B—Trail making test B; ADCS—ADCS mail-in cognitive function screening instrument; * Mann–Whitney U test.

**Table 6:** Summary statistics for neuropsychological test scores for education groups.

Variables	Education Groups						Test of Difference	
	≤12 Years			>12 Years			U-Test *	*p*-Value
	Med	Q_25_	Q_75_	Med	Q_25_	Q_75_		
MOCA/30	24	21	26	24	22	26	7702.0	0.014
CERAD learning trials, sum/30	16	13	19	18	14	21	7758.5	0.018
CERAD delayed recall/10	4	2	6	5	3	6	8218.0	0.095
CERAD word recognition: correct hits/10	9	7	10	9	8	10	8575.5	0.250
CERAD word recognition: correct rejections/10	10	9	10	10	9	10	8318.5	0.078
TMT A, time (s) *	55	43	74	50	40	62	7869.5	0.029
TMT A errors	0	0	0	0	0	0	8956.5	0.329
TMT B, time (s) *	140	101	218	125	94	176	7694.0	0.014
TMT B errors	1	0	2	1	0	2	9280.0	0.973
MINT/32	28	25	29	28	25	30	8605.5	0.286
Semantic fluency: animals (no. words)	14	10	17	15	12	19	7842.5	0.026
Semantic fluency: vegetables (no. words)	10	8	13	10	8	12	9271.0	0.963
Phonemic verbal fluency (no. words, sum of 3 trials)	24	18	32	29	22	38	6905.5	<0.001
ADCS/max score/x	3	1	5	2	1	4	8589.5	0.272

Med represents median. Q_25_ represents the 25th percentile and Q_75_ represents the 75th percentile. MOCA—Montreal Cognitive Assessment; CERAD—the Consortium to Establish a Registry in Alzheimer’s Disease; MINT—Multilingual Naming Test; TMT A—Trail making test A; TMT B—Trail making test B; ADCS—ADCS mail-in cognitive function screening instrument; * Mann–Whitney U test.

**Table 7:** Multiple regression models for neuropsychological test scores.

Neuropsychological Test Battery	Age	Education	Gender	Ethnicity
MOCA/30	−0.22	0.26	−0.57	2.73
	(−0.299, −0.146) **	(0.133, 0.390) **	(−1.305, 0.173)	(2.007, 3.448) **
CERAD learning trials, sum/30	−0.33	0.26	−1.05	2.09
	(−0.447, −0.223) **	(0.076, 0.452) **	(−2.129, 0.032)	(1.036, 3.144) **
CERAD delayed recall/10	−0.15	0.10	−0.93	0.21
	(−0.208, −0.095) **	(0.010, 0.200) *	(−1.478, −0.386) **	(−0.326, 0.739)
CERAD word recognition: correct hits/10	−0.13	0.11	−0.12	0.58
	(−0.173, −0.079) **	(0.031, 0.189) **	(−0.578, 0.330)	(0.134, 1.021) *
CERAD word recognition: correct rejections/10	−0.02	0.05	−0.07	0.12
	(−0.044, −0.001) *	(0.011, 0.082) *	(−0.270, 0.136)	(−0.080, 0.316)
TMT A, time (s)	1.19	−1.81	3.01	−13.44
	(0.507, 1.865) **	(−2.951, −0.665) **	(−3.555, 9.578)	(−19.847, −7.036) **
TMT A errors ^†^	0.00	−0.01	−0.02	−0.19
	(−0.006, 0.015)	(−0.031, 0.004)	(−0.124, 0.076)	(−0.291, −0.097) **
TMT B, time (s)	4.17	−5.45	8.38	−35.96
	(2.507, 5.837) **	(−8.256, −2.648) **	(−7.730, 24.489)	(−51.674, −20.249) **
TMT B errors ^†^	0.10	−0.07	−0.14	−0.58
	(0.057, 0.152) **	(−0.150, 0.010)	(−0.597, 0.324)	(−1.030, −0.132) *
MINT/32	−0.20	0.11	1.91	3.52
	(−0.295, −0.105) **	(−0.051, 0.269)	(0.995, 2.832) **	(2.624, 4.416) **
Semantic fluency: animals (no. words)	−0.15	0.22	−0.70	2.90
	(−0.270, −0.026) *	(0.018, 0.430) *	(−1.881, 0.484)	(1.745, 4.051) **
Semantic fluency: vegetables (no. words)	−0.16	−0.01	−1.74	1.74
	(−0.249, −0.076) **	(−0.160, 0.131)	(−2.575, −0.904) **	(0.928, 2.558) **
Phonemic verbal fluency (no. words. sum of 3 trials)	−0.54	0.94	−1.90	4.14
	(−0.789, −0.301) **	(0.533, 1.354) **	(−4.262, 0.457)	(1.838, 6.441) **
ADCS/max score/x ^†^	0.09	−0.08	−0.67	−0.43
	(0.024, 0.159) **	(−0.189, 0.039)	(−1.327, −0.017) *	(−1.073, 0.205)

Values are expressed as a beta-coefficient (95% confidence interval). MOCA—Montreal Cognitive Assessment; CERAD—the Consortium to Establish a Registry in Alzheimer’s Disease; MINT—Multilingual Naming Test; TMT A—Trail making test A; TMT B—Trail making test B; ADCS—ADCS mail-in cognitive function screening instrument; Gender was categorized into male and female. The reference variable is male. Education and Age were analyzed as a continuous variable. The reference for ethnicity was Russian and European category. * Indicates significance at *p* ≤ 0.05. ** Indicates significance at *p* ≤ 0.01. ^†^ Indicates log transformed.

## Data Availability

The data presented in this study may be available on request from the corresponding author (A.S.). The data are not publicly available due to restrictions, i.e., their containing information that could compromise the privacy of participants.
